# Supplementary material for: Evolution of the Spider Homeobox Gene Repertoire by Tandem and Whole Genome Duplication
Source: Mol Biol Evol. 2023 Nov 3;40(12):msad239. doi: 10.1093/molbev/msad239 (PMC10726417; doi:10.1093/molbev/msad239)
Supplement: msad239_Supplementary_Data [file msad239_supplementary_data.zip › FigureS3.pdf]

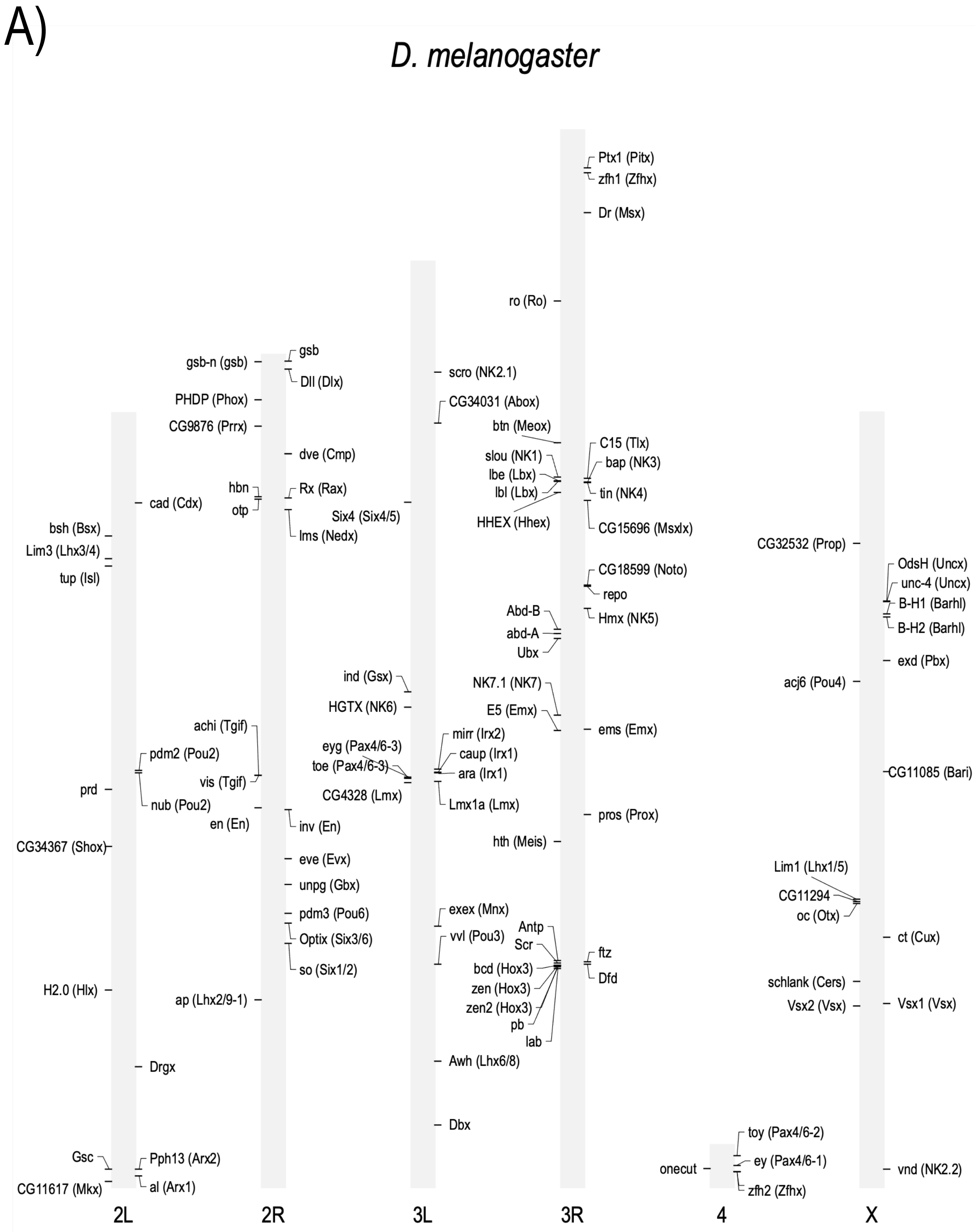
A)
D. melanogaster
Ptx1 (Pitx)
zfh1 (Zfhx)
Dr (Msx)
ro (Ro)
gsb-n (gsb)
gsb
Dll (Dlx)
scro (NK2.1)
PHDP (Phox)
CG34031 (Abox)
CG9876 (Prrx)
btn (Meox)
dve (Cmp)
C15 (Tlx)
slou (NK1)
bap (NK3)
lbe (Lbx)
hbn
lbl (Lbx)
Rx (Rax)
tin (NK4)
cad (Cdx)
otp
Six4 (Six4/5)
HHEX (Hhex)
bsh (Bsx)
lms (Nedx)
CG15696 (Msxlx)
Lim3 (Lhx3/4)
CG32532 (Prop)
tup (Isl)
OdsH (Uncx)
CG18599 (Noto)
unc-4 (Uncx)
repo
B-H1 (Barhl)
Abd-B
Hmx (NK5)
abd-A
B-H2 (Barhl)
Ubx
exd (Pbx)
ind (Gsx)
NK7.1 (NK7)
acj6 (Pou4)
HGTX (NK6)
E5 (Emx)
achi (Tgif)
ems (Emx)
mirr (Irx2)
pdm2 (Pou2)
eyg (Pax4/6-3)
caup (Irx1)
toe (Pax4/6-3)
ara (Irx1)
CG11085 (Bari)
vis (Tgif)
prd
CG4328 (Lmx)
nub (Pou2)
Lmx1a (Lmx)
pros (Prox)
en (En)
inv (En)
hth (Meis)
CG34367 (Shox)
eve (Evx)
Lim1 (Lhx1/5)
unpg (Gbx)
CG11294
exex (Mnx)
oc (Otx)
pdm3 (Pou6)
Antp
Optix (Six3/6)
vvl (Pou3)
ct (Cux)
Scr
ftz
so (Six1/2)
bcd (Hox3)
Dfd
zen (Hox3)
schlank (Cers)
H2.0 (Hlx)
ap (Lhx2/9-1)
zen2 (Hox3)
Vsx2 (Vsx)
Vsx1 (Vsx)
pb
lab
Awh (Lhx6/8)
Drgx
Dbx
toy (Pax4/6-2)
Gsc
Pph13 (Arx2)
onecut
ey (Pax4/6-1)
vnd (NK2.2)
CG11617 (Mkx)
al (Arx1)
zfh2 (Zfhx)
2L
2R
3L
3R
4
X

B)

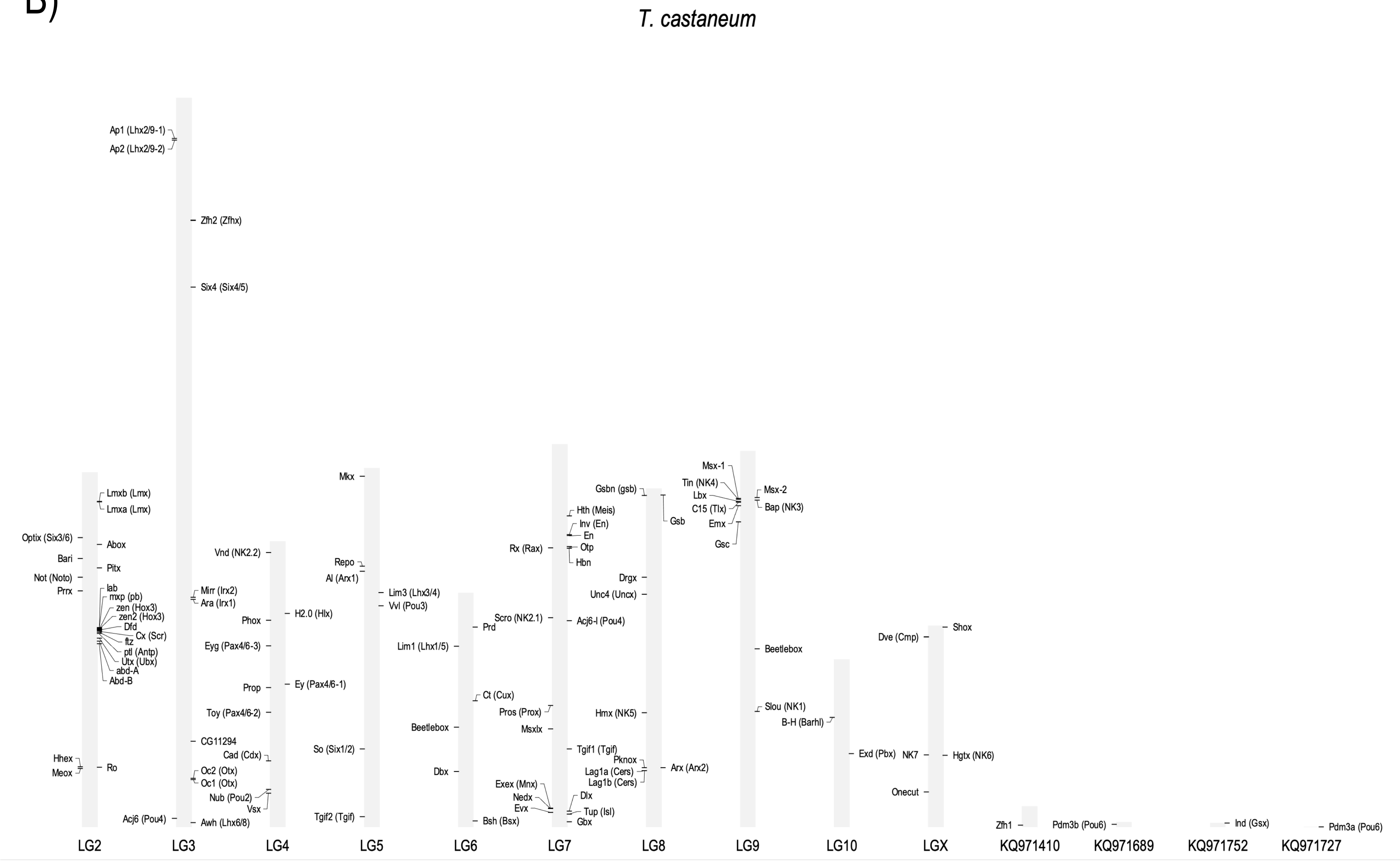
T. castaneum
LG2
Optix (Six3/6)
Bari
Not (Noto)
Prrx
Lmxb (Lmx)
Lmxa (Lmx)
Abox
Pitx
lab
mxp (pb)
zen (Hox3)
zen2 (Hox3)
Dfd
Cx (Scr)
ftz
ptl (Antp)
Utx (Ubx)
abd-A
Abd-B
Hhex
Meox
Ro
LG3
Ap1 (Lhx2/9-1)
Ap2 (Lhx2/9-2)
Zfh2 (Zfhx)
Six4 (Six4/5)
Mirr (Irx2)
Ara (Irx1)
CG11294
Oc2 (Otx)
Oc1 (Otx)
Acj6 (Pou4)
Awh (Lhx6/8)
LG4
Vnd (NK2.2)
Phox
H2.0 (Hlx)
Eyg (Pax4/6-3)
Prop
Ey (Pax4/6-1)
Toy (Pax4/6-2)
Cad (Cdx)
Nub (Pou2)
Vsx
LG5
Mkx
Repo
Al (Arx1)
Lim3 (Lhx3/4)
Vvl (Pou3)
So (Six1/2)
Tgif2 (Tgif)
LG6
Prd
Lim1 (Lhx1/5)
Ct (Cux)
Beetlebox
Dbx
Bsh (Bsx)
LG7
Rx (Rax)
Hth (Meis)
Inv (En)
En
Otp
Hbn
Scro (NK2.1)
Acj6-l (Pou4)
Pros (Prox)
Msxlx
Tgif1 (Tgif)
Exex (Mnx)
Nedx
Evx
Dlx
Tup (Isl)
Gbx
LG8
Gsbn (gsb)
Gsb
Drgx
Unc4 (Uncx)
Hmx (NK5)
Pknox
Lag1a (Cers)
Lag1b (Cers)
Arx (Arx2)
LG9
Msx-1
Tin (NK4)
Lbx
C15 (Tlx)
Emx
Gsc
Msx-2
Bap (NK3)
Beetlebox
Slou (NK1)
B-H (Barhl)
LG10
Exd (Pbx)
LGX
Dve (Cmp)
Shox
NK7
Hgtx (NK6)
Onecut
KQ971410
Zfh1
KQ971689
Pdm3b (Pou6)
KQ971752
Ind (Gsx)
KQ971727
Pdm3a (Pou6)

C)

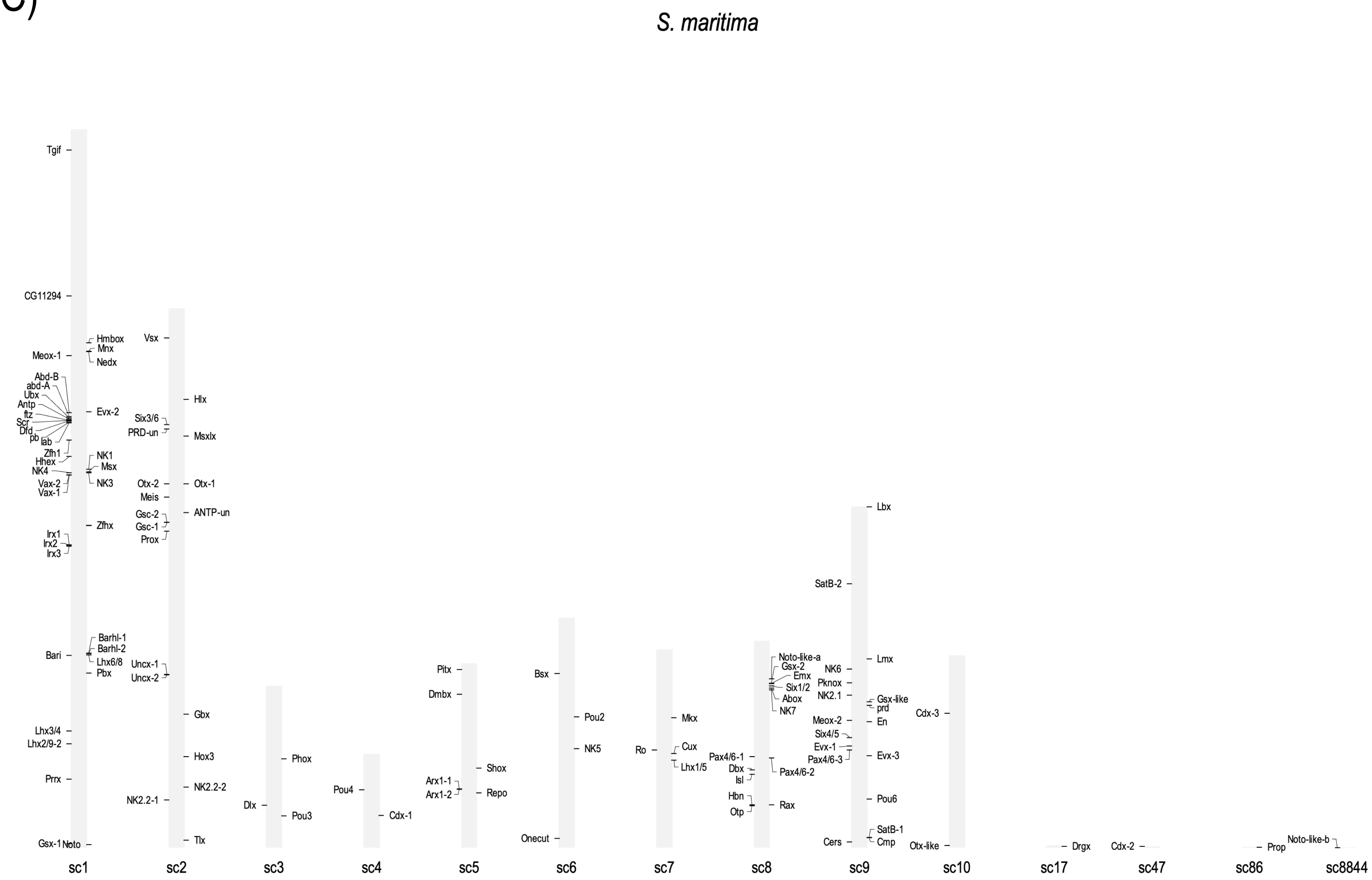
S. maritima
sc1
Tgif
CG11294
Meox-1
Hmbox
Mnx
Nedx
Abd-B
abd-A
Ubx
Antp
ftz
Scr
Dfd
pb
lab
Evx-2
Zfh1
Hhex
NK4
NK1
Msx
NK3
Vax-2
Vax-1
Zfhx
Irx1
Irx2
Irx3
Barhl-1
Barhl-2
Bari
Lhx6/8
Pbx
Lhx3/4
Lhx2/9-2
Prrx
Gsx-1
Noto
sc2
Vsx
Hlx
Six3/6
PRD-un
Msxlx
Otx-2
Otx-1
Meis
ANTP-un
Gsc-2
Gsc-1
Prox
Uncx-1
Uncx-2
Gbx
Hox3
NK2.2-2
NK2.2-1
Tlx
sc3
Phox
Dlx
Pou3
sc4
Pou4
Cdx-1
sc5
Pitx
Dmbx
Shox
Arx1-1
Arx1-2
Repo
sc6
Bsx
Pou2
NK5
Onecut
sc7
Mkx
Ro
Cux
Lhx1/5
sc8
Noto-like-a
Gsx-2
Emx
Six1/2
Abox
NK7
Pax4/6-1
Dbx
Isl
Pax4/6-2
Hbn
Otp
Rax
sc9
Lbx
SatB-2
Lmx
NK6
Pknox
NK2.1
Gsx-like
prd
Meox-2
En
Six4/5
Evx-1
Pax4/6-3
Evx-3
Pou6
SatB-1
Cers
Cmp
sc10
Cdx-3
Otx-like
sc17
Drgx
sc47
Cdx-2
sc86
Prop
sc8844
Noto-like-b

D)

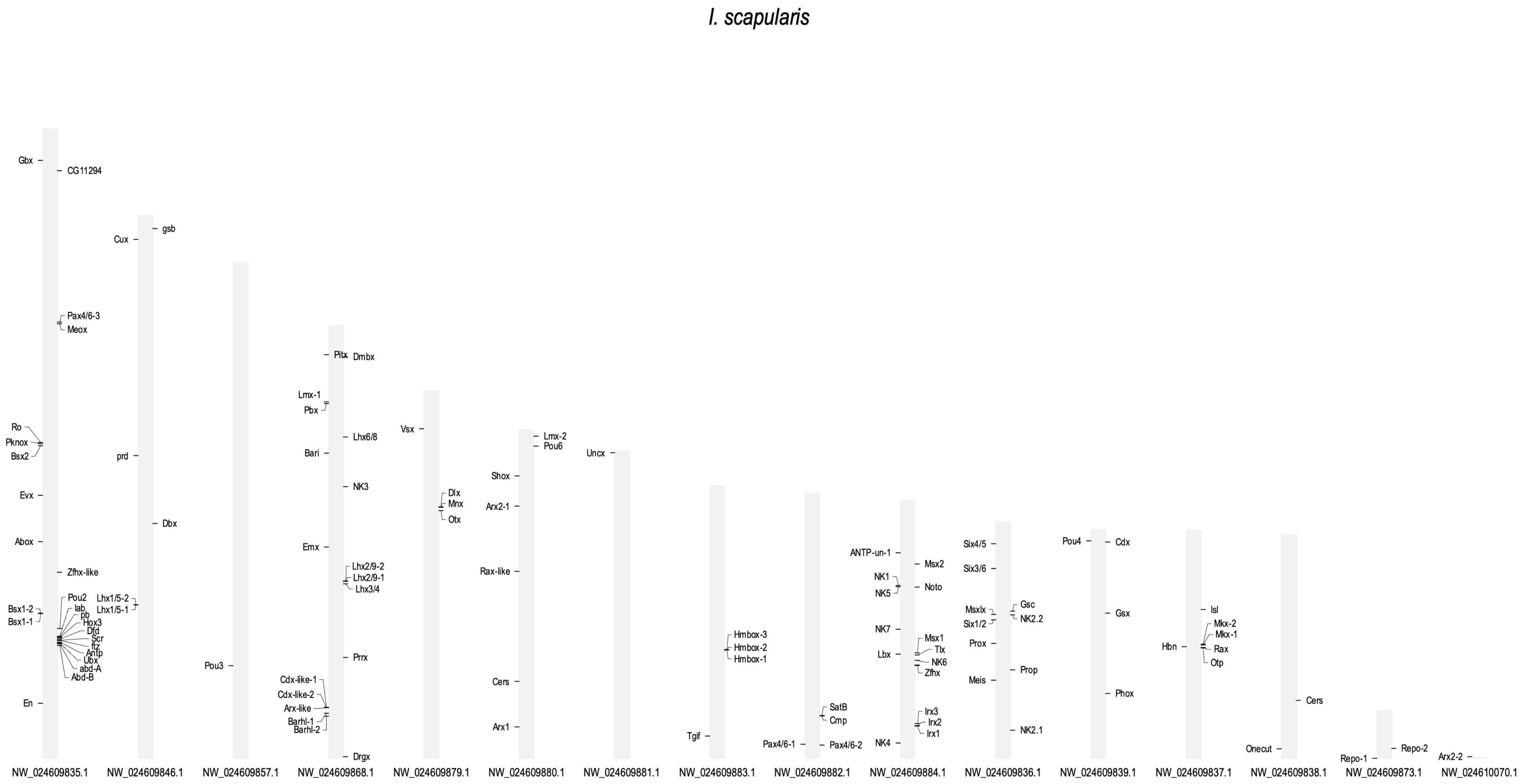
I. scapularis
Gbx
CG11294
Pax4/6-3
Meox
Ro
Pknox
Bsx2
Evx
Abox
Zfhx-like
Bsx1-2
Bsx1-1
Pou2
lab
pb
Hox3
Dfd
Scr
ftz
Antp
Ubx
abd-A
Abd-B
En
NW_024609835.1
Cux
gsb
prd
Dbx
Lhx1/5-2
Lhx1/5-1
NW_024609846.1
Pou3
NW_024609857.1
Pitx
Dmbx
Lmx-1
Pbx
Lhx6/8
Bari
NK3
Emx
Lhx2/9-2
Lhx2/9-1
Lhx3/4
Prrx
Cdx-like-1
Cdx-like-2
Arx-like
Barhl-1
Barhl-2
Drgx
NW_024609868.1
Vsx
Dlx
Mnx
Otx
NW_024609879.1
Lmx-2
Pou6
Shox
Arx2-1
Rax-like
Cers
Arx1
NW_024609880.1
Uncx
NW_024609881.1
Hmbox-3
Hmbox-2
Hmbox-1
Tgif
NW_024609883.1
SatB
Cmp
Pax4/6-1
Pax4/6-2
NW_024609882.1
ANTP-un-1
Msx2
NK1
NK5
Noto
NK7
Msx1
Tlx
Lbx
NK6
Zfhx
Irx3
Irx2
Irx1
NK4
NW_024609884.1
Six4/5
Six3/6
Msxlx
Gsc
Six1/2
NK2.2
Prox
Prop
Meis
NK2.1
NW_024609836.1
Pou4
Cdx
Gsx
Phox
NW_024609839.1
Isl
Mkx-2
Mkx-1
Hbn
Rax
Otp
NW_024609837.1
Cers
Onecut
NW_024609838.1
Repo-2
Repo-1
NW_024609873.1
Arx2-2
NW_024610070.1

E)

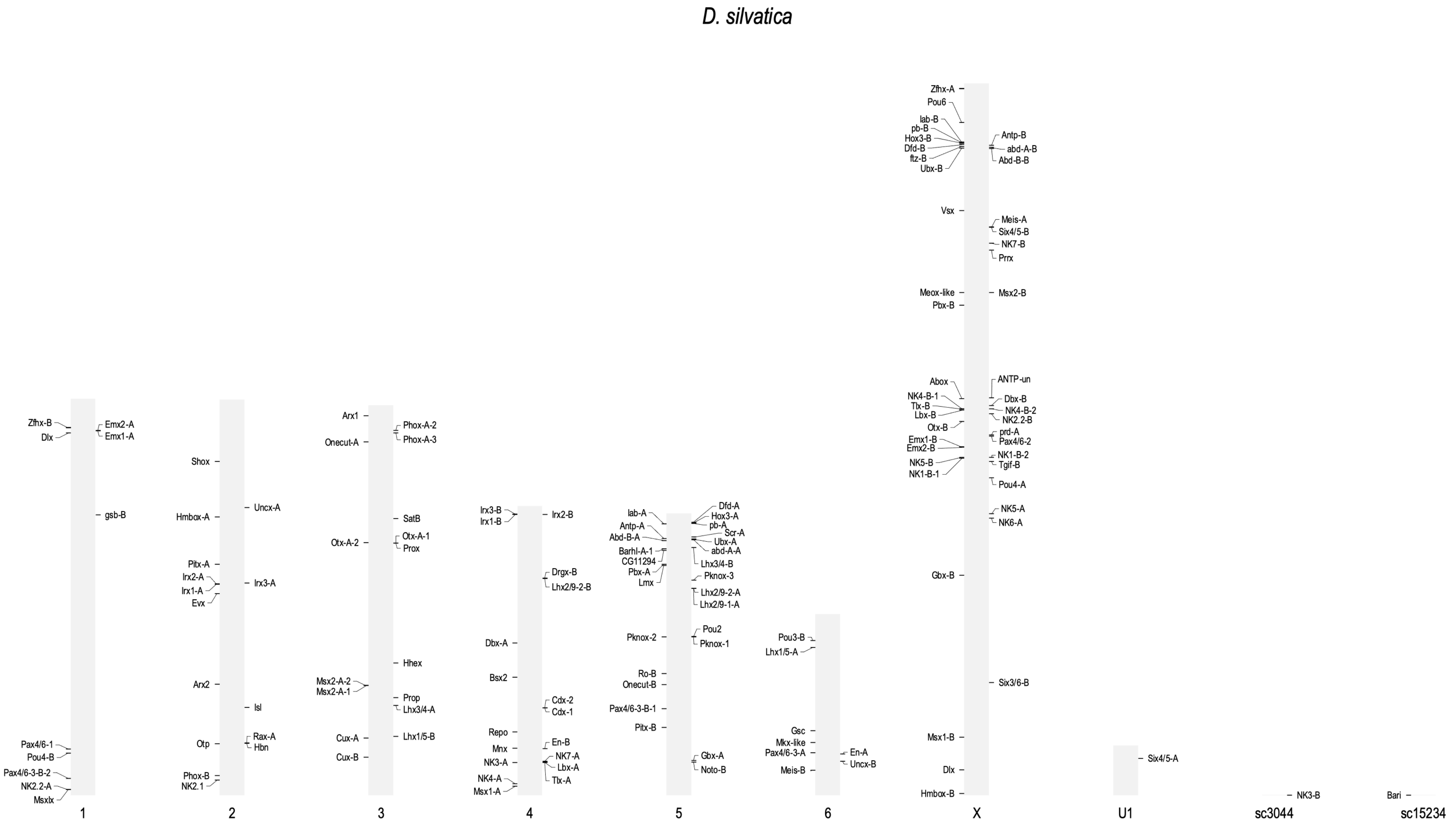
D. silvatica
Zfhx-B
Dlx
Emx2-A
Emx1-A
gsb-B
Pax4/6-1
Pou4-B
Pax4/6-3-B-2
NK2.2-A
Msxlx
1
Shox
Hmbox-A
Uncx-A
Pitx-A
Irx2-A
Irx1-A
Evx
Irx3-A
Arx2
Isl
Otp
Rax-A
Hbn
Phox-B
NK2.1
2
Arx1
Onecut-A
Phox-A-2
Phox-A-3
SatB
Otx-A-2
Otx-A-1
Prox
Hhex
Msx2-A-2
Msx2-A-1
Prop
Lhx3/4-A
Cux-A
Cux-B
Lhx1/5-B
3
Irx3-B
Irx1-B
Irx2-B
Drgx-B
Lhx2/9-2-B
Dbx-A
Bsx2
Cdx-2
Cdx-1
Repo
Mnx
NK3-A
NK4-A
Msx1-A
En-B
NK7-A
Lbx-A
Tlx-A
4
lab-A
Antp-A
Abd-B-A
Barhl-A-1
CG11294
Pbx-A
Lmx
Dfd-A
Hox3-A
pb-A
Scr-A
Ubx-A
abd-A-A
Lhx3/4-B
Pknox-3
Lhx2/9-2-A
Lhx2/9-1-A
Pknox-2
Pou2
Pknox-1
Ro-B
Onecut-B
Pax4/6-3-B-1
Pitx-B
Gbx-A
Noto-B
5
Pou3-B
Lhx1/5-A
Gsc
Mkx-like
Pax4/6-3-A
Meis-B
En-A
Uncx-B
6
Zfhx-A
Pou6
lab-B
pb-B
Hox3-B
Dfd-B
ftz-B
Ubx-B
Antp-B
abd-A-B
Abd-B-B
Vsx
Meis-A
Six4/5-B
NK7-B
Prrx
Meox-like
Pbx-B
Msx2-B
Abox
NK4-B-1
Tlx-B
Lbx-B
Otx-B
Emx1-B
Emx2-B
NK5-B
NK1-B-1
ANTP-un
Dbx-B
NK4-B-2
NK2.2-B
prd-A
Pax4/6-2
NK1-B-2
Tgif-B
Pou4-A
NK5-A
NK6-A
Gbx-B
Six3/6-B
Msx1-B
Dlx
Hmbox-B
X
Six4/5-A
U1
NK3-B
sc3044
Bari
sc15234

F)

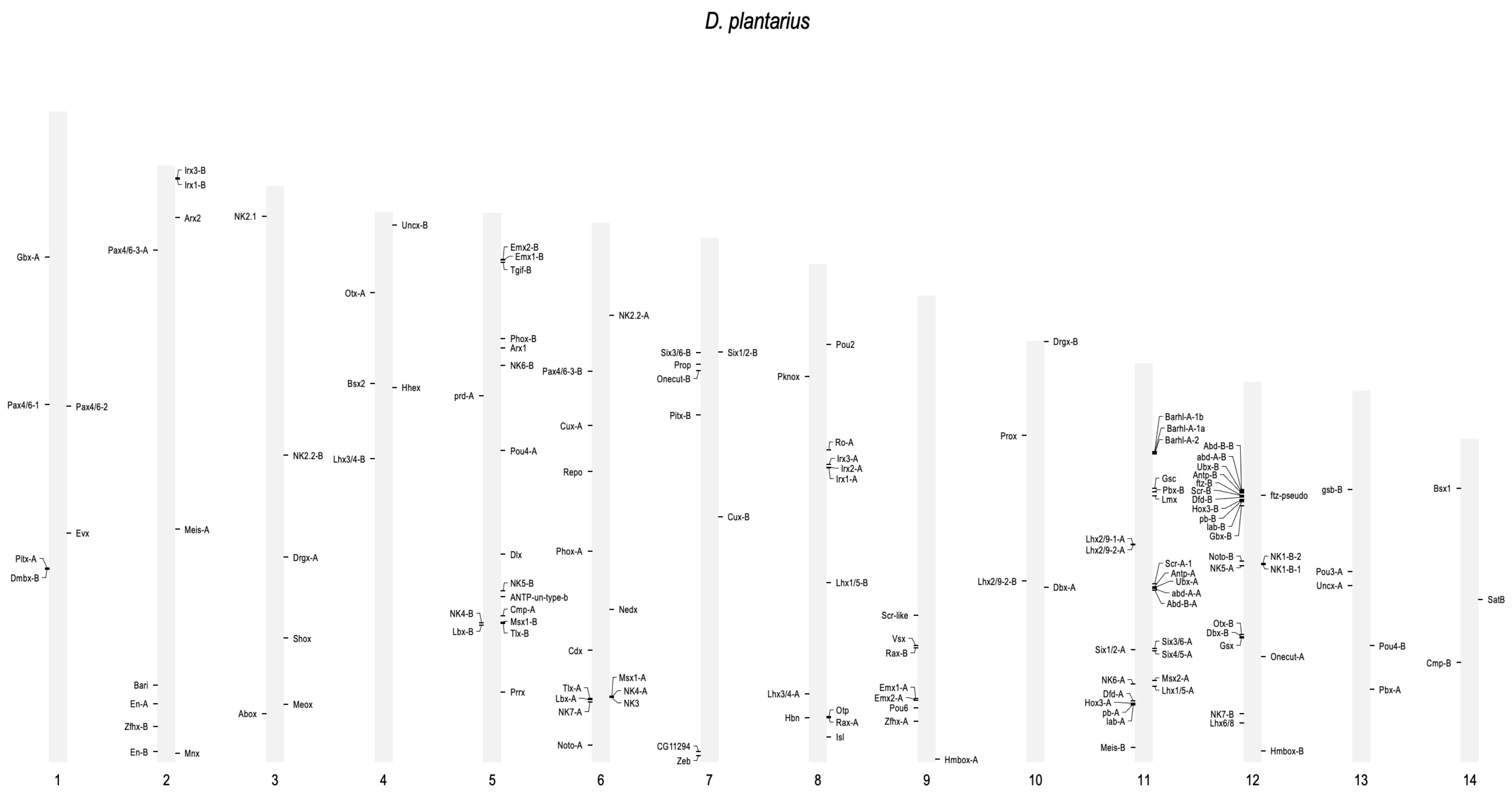
D. plantarius
1
Gbx-A
Pax4/6-1
Pax4/6-2
Evx
Pitx-A
Dmbx-B
2
Irx3-B
Irx1-B
Arx2
Pax4/6-3-A
Meis-A
Bari
En-A
Zfhx-B
En-B
Mnx
3
NK2.1
NK2.2-B
Drgx-A
Shox
Meox
Abox
4
Uncx-B
Otx-A
Bsx2
Hhex
Lhx3/4-B
5
Emx2-B
Emx1-B
Tgif-B
Phox-B
Arx1
NK6-B
prd-A
Pou4-A
Dlx
NK5-B
ANTP-un-type-b
Cmp-A
Msx1-B
Tlx-B
NK4-B
Lbx-B
Prrx
6
NK2.2-A
Pax4/6-3-B
Cux-A
Repo
Phox-A
Nedx
Cdx
Msx1-A
NK4-A
NK3
Tlx-A
Lbx-A
NK7-A
Noto-A
7
Six3/6-B
Prop
Onecut-B
Six1/2-B
Pitx-B
Cux-B
CG11294
Zeb
8
Pou2
Pknox
Ro-A
Irx3-A
Irx2-A
Irx1-A
Lhx1/5-B
Lhx3/4-A
Otp
Rax-A
Hbn
Isl
9
Scr-like
Vsx
Rax-B
Emx1-A
Emx2-A
Pou6
Zfhx-A
Hmbox-A
10
Drgx-B
Prox
Lhx2/9-2-B
Dbx-A
11
Barhl-A-1b
Barhl-A-1a
Barhl-A-2
Gsc
Pbx-B
Lmx
Lhx2/9-1-A
Lhx2/9-2-A
Scr-A-1
Antp-A
Ubx-A
abd-A-A
Abd-B-A
Six1/2-A
Six3/6-A
Six4/5-A
NK6-A
Msx2-A
Lhx1/5-A
Dfd-A
Hox3-A
pb-A
lab-A
Meis-B
12
Abd-B-B
abd-A-B
Ubx-B
Antp-B
ftz-B
Scr-B
Dfd-B
Hox3-B
pb-B
lab-B
Gbx-B
ftz-pseudo
Noto-B
NK5-A
NK1-B-2
NK1-B-1
Otx-B
Dbx-B
Gsx
Onecut-A
NK7-B
Lhx6/8
Hmbox-B
13
gsb-B
Pou3-A
Uncx-A
Pou4-B
Pbx-A
14
Bsx1
SatB
Cmp-B

G)

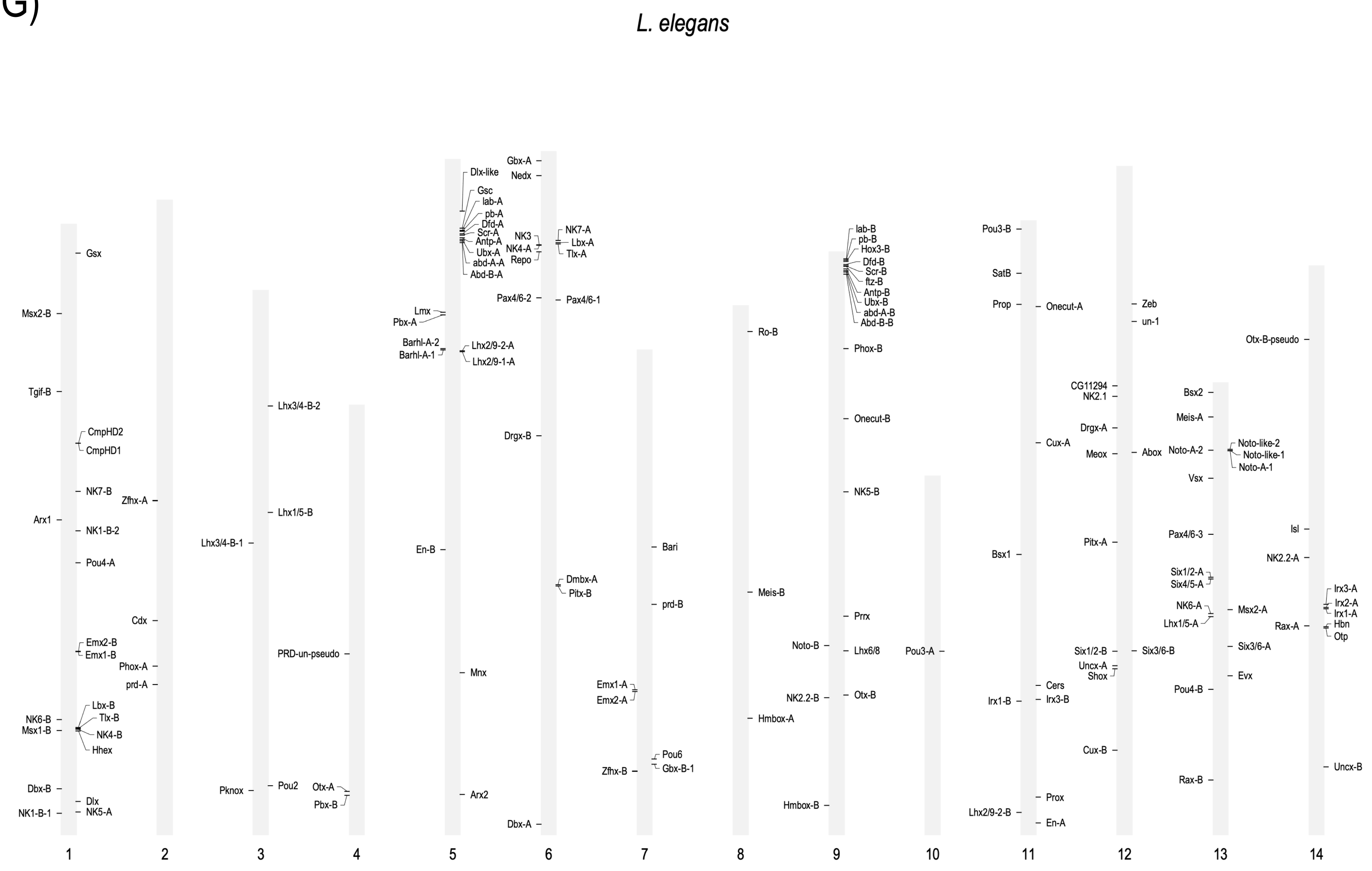
L. elegans
1
Gsx
Msx2-B
Tgif-B
CmpHD2
CmpHD1
NK7-B
Arx1
NK1-B-2
Pou4-A
Emx2-B
Emx1-B
Lbx-B
Tlx-B
NK6-B
Msx1-B
NK4-B
Hhex
Dbx-B
Dlx
NK1-B-1
NK5-A
2
Zfhx-A
Cdx
Phox-A
prd-A
3
Lhx3/4-B-2
Lhx1/5-B
Lhx3/4-B-1
Pou2
Pknox
4
PRD-un-pseudo
Otx-A
Pbx-B
5
Dlx-like
Gsc
lab-A
pb-A
Dfd-A
Scr-A
Antp-A
Ubx-A
abd-A-A
Abd-B-A
Lmx
Pbx-A
Barhl-A-2
Barhl-A-1
Lhx2/9-2-A
Lhx2/9-1-A
En-B
Mnx
Arx2
6
Gbx-A
Nedx
NK3
NK4-A
Repo
NK7-A
Lbx-A
Tlx-A
Pax4/6-2
Pax4/6-1
Drgx-B
Dmbx-A
Pitx-B
Dbx-A
7
Bari
prd-B
Emx1-A
Emx2-A
Pou6
Gbx-B-1
Zfhx-B
8
Ro-B
Meis-B
Hmbox-A
9
lab-B
pb-B
Hox3-B
Dfd-B
Scr-B
ftz-B
Antp-B
Ubx-B
abd-A-B
Abd-B-B
Phox-B
Onecut-B
NK5-B
Prrx
Noto-B
Lhx6/8
NK2.2-B
Otx-B
Hmbox-B
10
Pou3-A
11
Pou3-B
SatB
Prop
Onecut-A
Cux-A
Bsx1
Cers
Irx1-B
Irx3-B
Prox
Lhx2/9-2-B
En-A
12
Zeb
un-1
CG11294
NK2.1
Drgx-A
Meox
Abox
Pitx-A
Six1/2-B
Six3/6-B
Uncx-A
Shox
Cux-B
13
Bsx2
Meis-A
Noto-A-2
Noto-like-2
Noto-like-1
Noto-A-1
Vsx
Pax4/6-3
Six1/2-A
Six4/5-A
NK6-A
Msx2-A
Lhx1/5-A
Six3/6-A
Evx
Pou4-B
Rax-B
14
Otx-B-pseudo
Isl
NK2.2-A
Irx3-A
Irx2-A
Irx1-A
Rax-A
Hbn
Otp
Uncx-B

H)

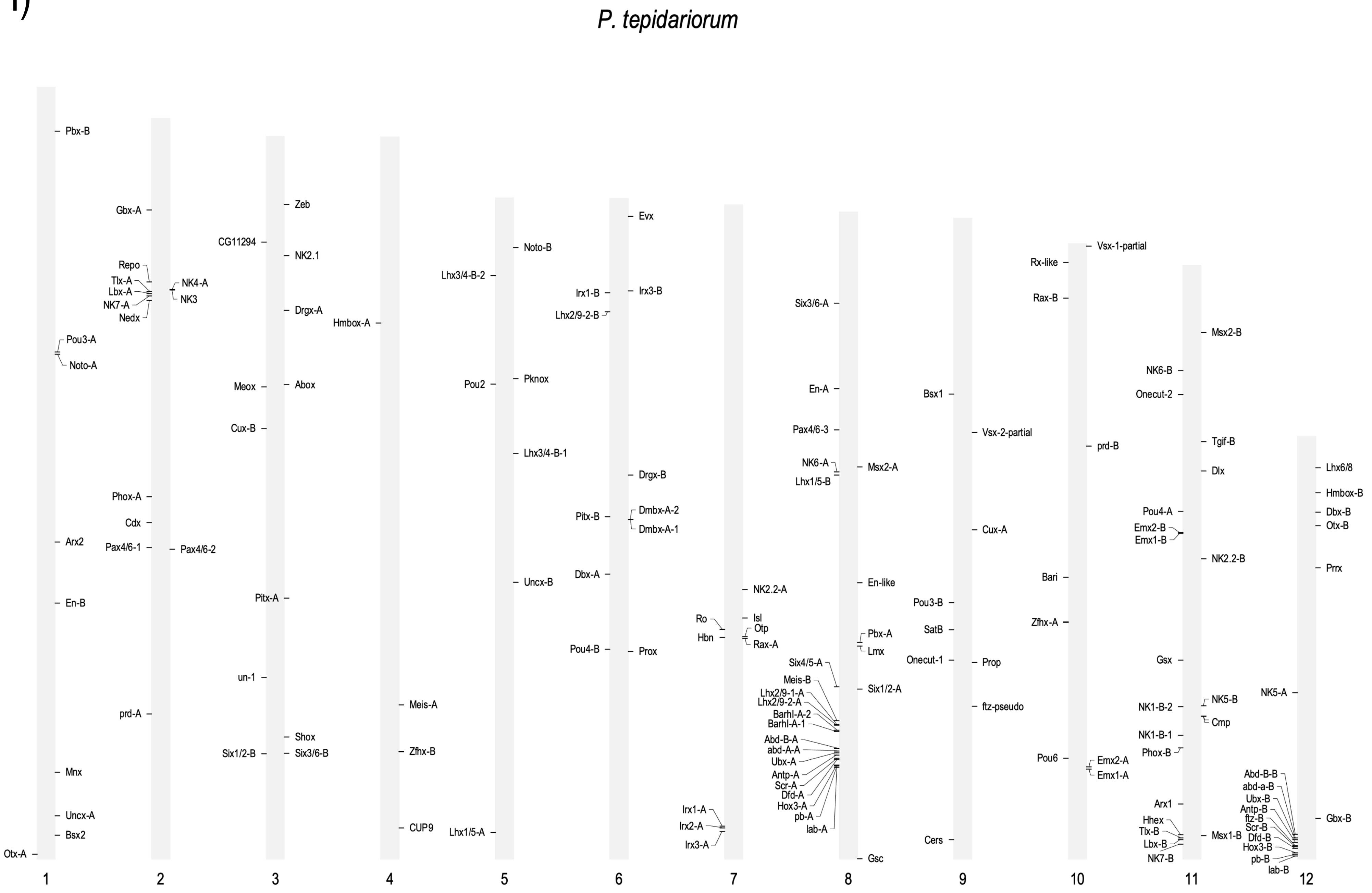
P. tepidariorum
1
Pbx-B
Pou3-A
Noto-A
Arx2
En-B
Mnx
Uncx-A
Bsx2
Otx-A
2
Gbx-A
Repo
Tlx-A
Lbx-A
NK7-A
Nedx
NK4-A
NK3
Phox-A
Cdx
Pax4/6-1
Pax4/6-2
prd-A
3
Zeb
CG11294
NK2.1
Drgx-A
Meox
Abox
Cux-B
Pitx-A
un-1
Shox
Six1/2-B
Six3/6-B
4
Hmbox-A
Meis-A
Zfhx-B
CUP9
5
Noto-B
Lhx3/4-B-2
Pou2
Pknox
Lhx3/4-B-1
Uncx-B
Lhx1/5-A
6
Evx
Irx1-B
Irx3-B
Lhx2/9-2-B
Drgx-B
Pitx-B
Dmbx-A-2
Dmbx-A-1
Dbx-A
Pou4-B
Prox
7
NK2.2-A
Ro
Isl
Otp
Hbn
Rax-A
Irx1-A
Irx2-A
Irx3-A
8
Six3/6-A
En-A
Pax4/6-3
NK6-A
Msx2-A
Lhx1/5-B
En-like
Pbx-A
Lmx
Six4/5-A
Meis-B
Six1/2-A
Lhx2/9-1-A
Lhx2/9-2-A
Barhl-A-2
Barhl-A-1
Abd-B-A
abd-A-A
Ubx-A
Antp-A
Scr-A
Dfd-A
Hox3-A
pb-A
lab-A
Gsc
9
Bsx1
Vsx-2-partial
Cux-A
Pou3-B
SatB
Onecut-1
Prop
ftz-pseudo
Cers
10
Vsx-1-partial
Rx-like
Rax-B
prd-B
Bari
Zfhx-A
Pou6
Emx2-A
Emx1-A
11
Msx2-B
NK6-B
Onecut-2
Tgif-B
Dlx
Pou4-A
Emx2-B
Emx1-B
NK2.2-B
Gsx
NK1-B-2
NK5-B
Cmp
NK1-B-1
Phox-B
Arx1
Hhex
Tlx-B
Msx1-B
Lbx-B
NK7-B
12
Lhx6/8
Hmbox-B
Dbx-B
Otx-B
Prrx
NK5-A
Abd-B-B
abd-a-B
Ubx-B
Antp-B
ftz-B
Scr-B
Dfd-B
Hox3-B
pb-B
lab-B
Gbx-B

l)

H. graminicola

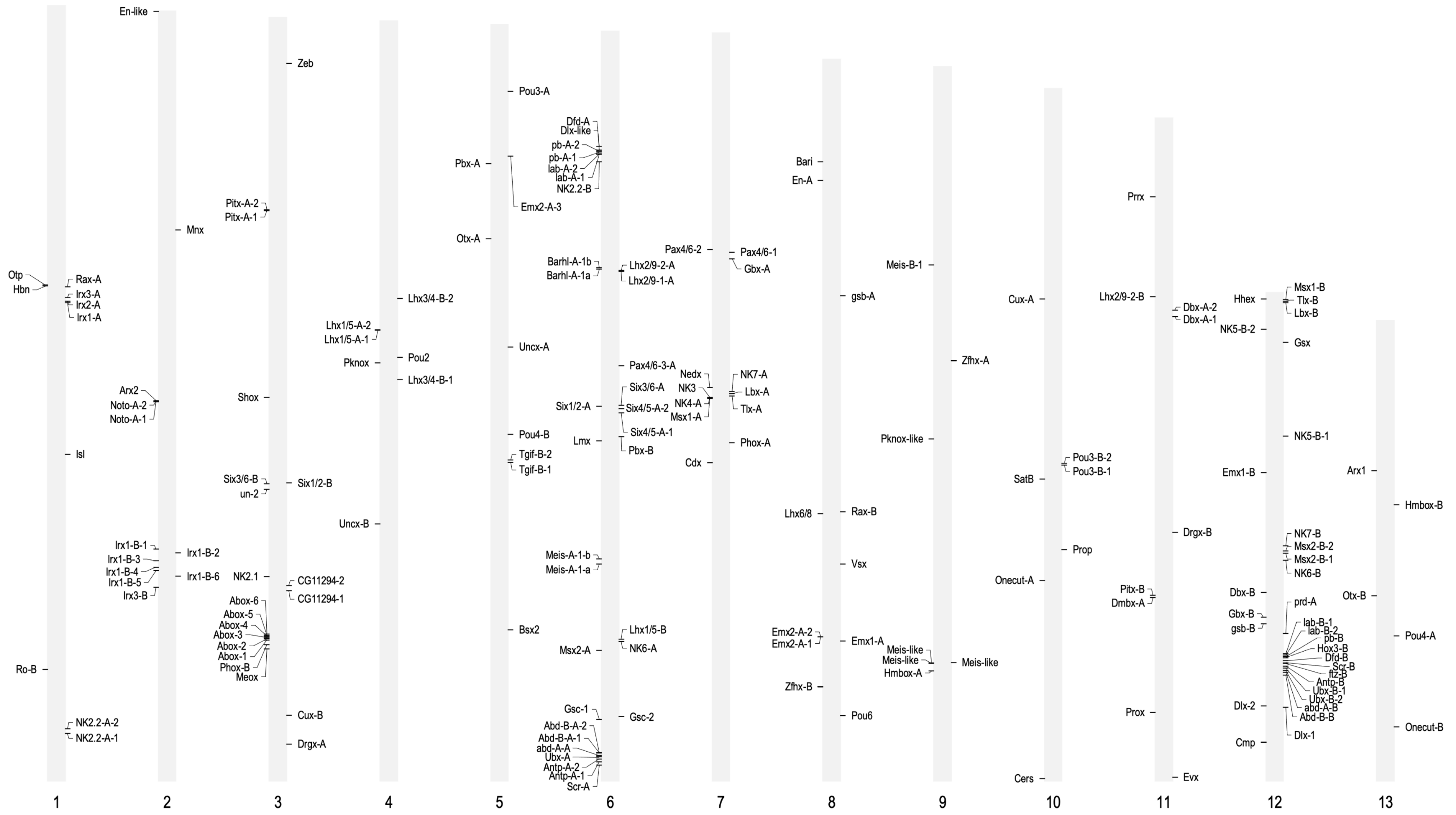

J)

A. bruennichi

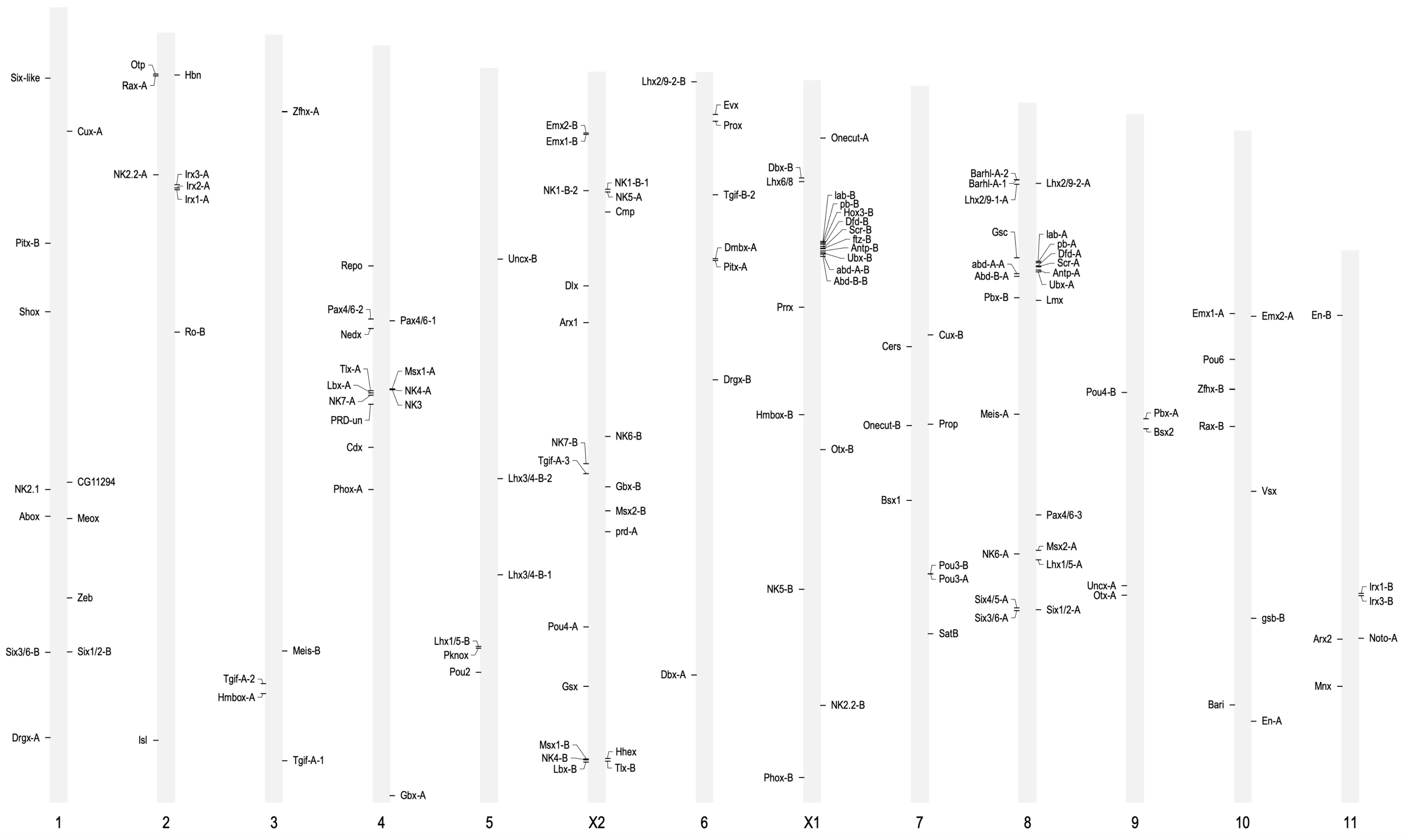

K)

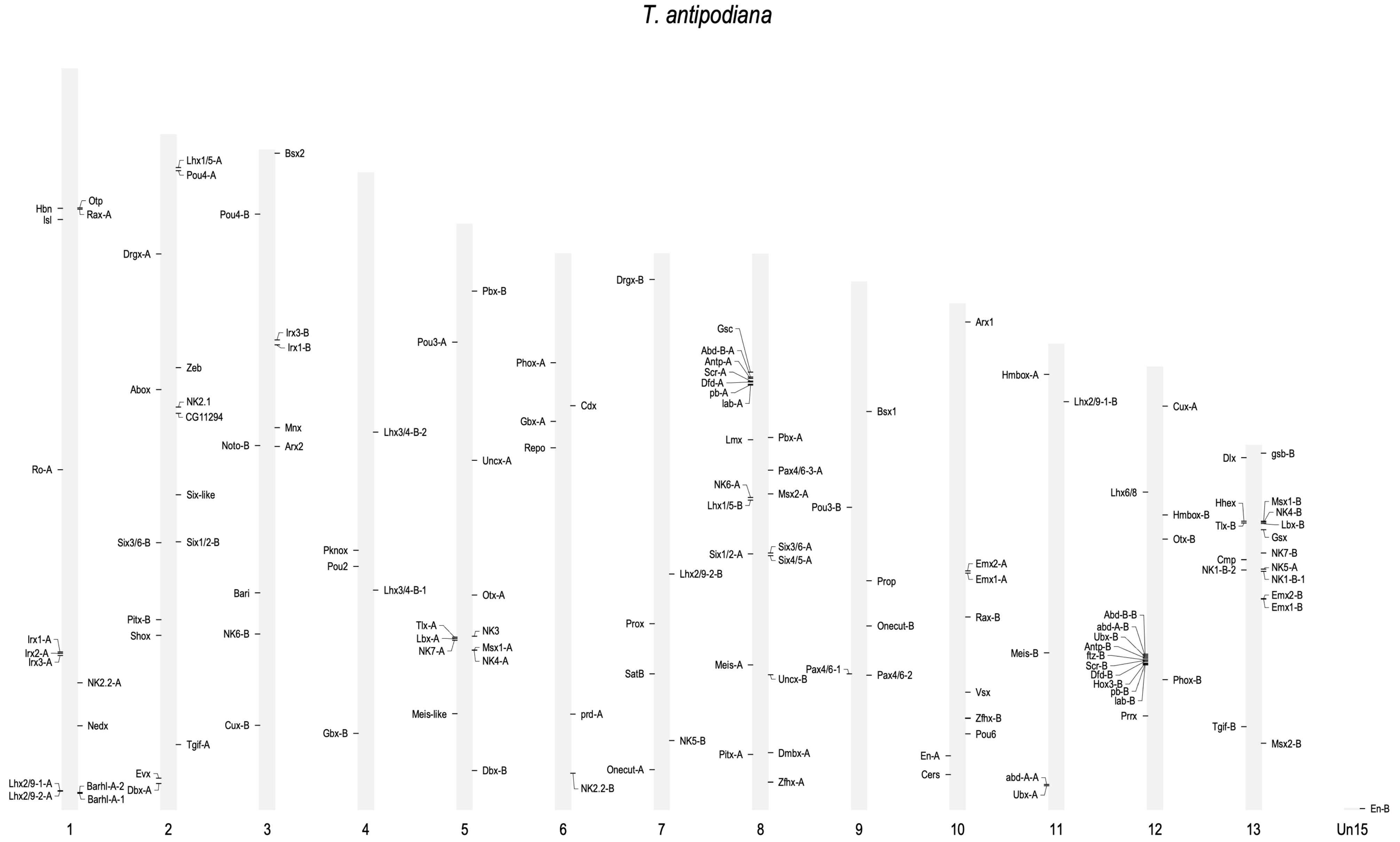
T. antipodiana
1
Hbn
Isl
Otp
Rax-A
Ro-A
Irx1-A
Irx2-A
Irx3-A
NK2.2-A
Nedx
Lhx2/9-1-A
Lhx2/9-2-A
Barhl-A-2
Barhl-A-1
2
Lhx1/5-A
Pou4-A
Drgx-A
Zeb
Abox
NK2.1
CG11294
Six-like
Six3/6-B
Six1/2-B
Pitx-B
Shox
Tgif-A
Evx
Dbx-A
3
Bsx2
Pou4-B
Irx3-B
Irx1-B
Mnx
Noto-B
Arx2
Bari
NK6-B
Cux-B
4
Lhx3/4-B-2
Pknox
Pou2
Lhx3/4-B-1
Gbx-B
5
Pbx-B
Pou3-A
Uncx-A
Otx-A
Tlx-A
Lbx-A
NK7-A
NK3
Msx1-A
NK4-A
Meis-like
Dbx-B
6
Phox-A
Cdx
Gbx-A
Repo
prd-A
NK2.2-B
7
Drgx-B
Lhx2/9-2-B
Prox
SatB
NK5-B
Onecut-A
8
Gsc
Abd-B-A
Antp-A
Scr-A
Dfd-A
pb-A
lab-A
Lmx
Pbx-A
Pax4/6-3-A
NK6-A
Msx2-A
Lhx1/5-B
Six1/2-A
Six3/6-A
Six4/5-A
Meis-A
Uncx-B
Pitx-A
Dmbx-A
Zfhx-A
9
Bsx1
Pou3-B
Prop
Onecut-B
Pax4/6-1
Pax4/6-2
10
Arx1
Emx2-A
Emx1-A
Rax-B
Vsx
Zfhx-B
Pou6
En-A
Cers
11
Hmbox-A
Lhx2/9-1-B
Meis-B
abd-A-A
Ubx-A
12
Cux-A
Lhx6/8
Hmbox-B
Otx-B
Abd-B-B
abd-A-B
Ubx-B
Antp-B
ftz-B
Scr-B
Dfd-B
Hox3-B
pb-B
lab-B
Phox-B
Prrx
13
Dlx
gsb-B
Hhex
Msx1-B
NK4-B
Tlx-B
Lbx-B
Gsx
NK7-B
Cmp
NK5-A
NK1-B-2
NK1-B-1
Emx2-B
Emx1-B
Tgif-B
Msx2-B
Un15
En-B

L)

T. clavata

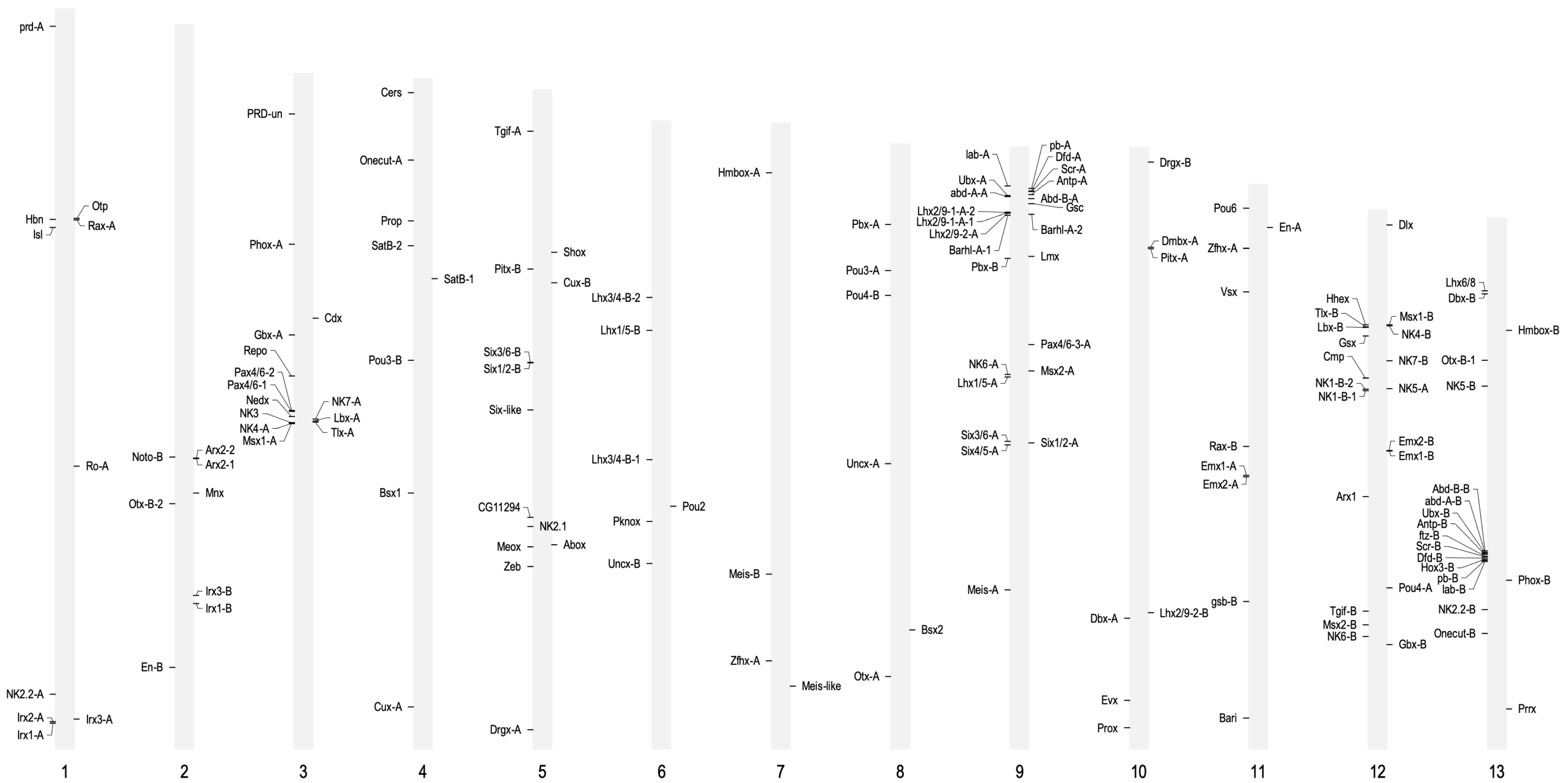
prd-A
Hbn
Isl
Otp
Rax-A
Ro-A
NK2.2-A
Irx2-A
Irx1-A
Irx3-A
Noto-B
Otx-B-2
En-B
Arx2-2
Arx2-1
Mnx
Irx3-B
Irx1-B
PRD-un
Phox-A
Gbx-A
Repo
Pax4/6-2
Pax4/6-1
Nedx
NK3
NK4-A
Msx1-A
Cdx
NK7-A
Lbx-A
Tlx-A
Cers
Onecut-A
Prop
SatB-2
Pou3-B
Bsx1
Cux-A
SatB-1
Tgif-A
Pitx-B
Six3/6-B
Six1/2-B
Six-like
CG11294
Meox
Zeb
Drgx-A
Shox
Cux-B
NK2.1
Abox
Lhx3/4-B-2
Lhx1/5-B
Lhx3/4-B-1
Pknox
Uncx-B
Pou2
Hmbox-A
Meis-B
Zfhx-A
Meis-like
Pbx-A
Pou3-A
Pou4-B
Uncx-A
Otx-A
Bsx2
lab-A
Ubx-A
abd-A-A
Lhx2/9-1-A-2
Lhx2/9-1-A-1
Lhx2/9-2-A
Barhl-A-1
Pbx-B
NK6-A
Lhx1/5-A
Six3/6-A
Six4/5-A
Meis-A
pb-A
Dfd-A
Scr-A
Antp-A
Abd-B-A
Gsc
Barhl-A-2
Lmx
Pax4/6-3-A
Msx2-A
Six1/2-A
Dbx-A
Evx
Prox
Drgx-B
Dmbx-A
Pitx-A
Lhx2/9-2-B
Pou6
Zfhx-A
Vsx
Rax-B
Emx1-A
Emx2-A
gsb-B
Bari
En-A
Hhex
Tlx-B
Lbx-B
Gsx
Cmp
NK1-B-2
NK1-B-1
Arx1
Tgif-B
Msx2-B
NK6-B
Dlx
Msx1-B
NK4-B
NK7-B
NK5-A
Emx2-B
Emx1-B
Pou4-A
Gbx-B
Lhx6/8
Dbx-B
Otx-B-1
NK5-B
Abd-B-B
abd-A-B
Ubx-B
Antp-B
ftz-B
Scr-B
Dfd-B
Hox3-B
pb-B
lab-B
NK2.2-B
Onecut-B
Hmbox-B
Phox-B
Prrx
1
2
3
4
5
6
7
8
9
10
11
12
13
