# Supplementary figures and images for: Evolution of the Spider Homeobox Gene Repertoire by Tandem and Whole Genome Duplication
Source: Mol Biol Evol. 2023 Nov 3;40(12):msad239. doi: 10.1093/molbev/msad239 (PMC10726417; doi:10.1093/molbev/msad239)

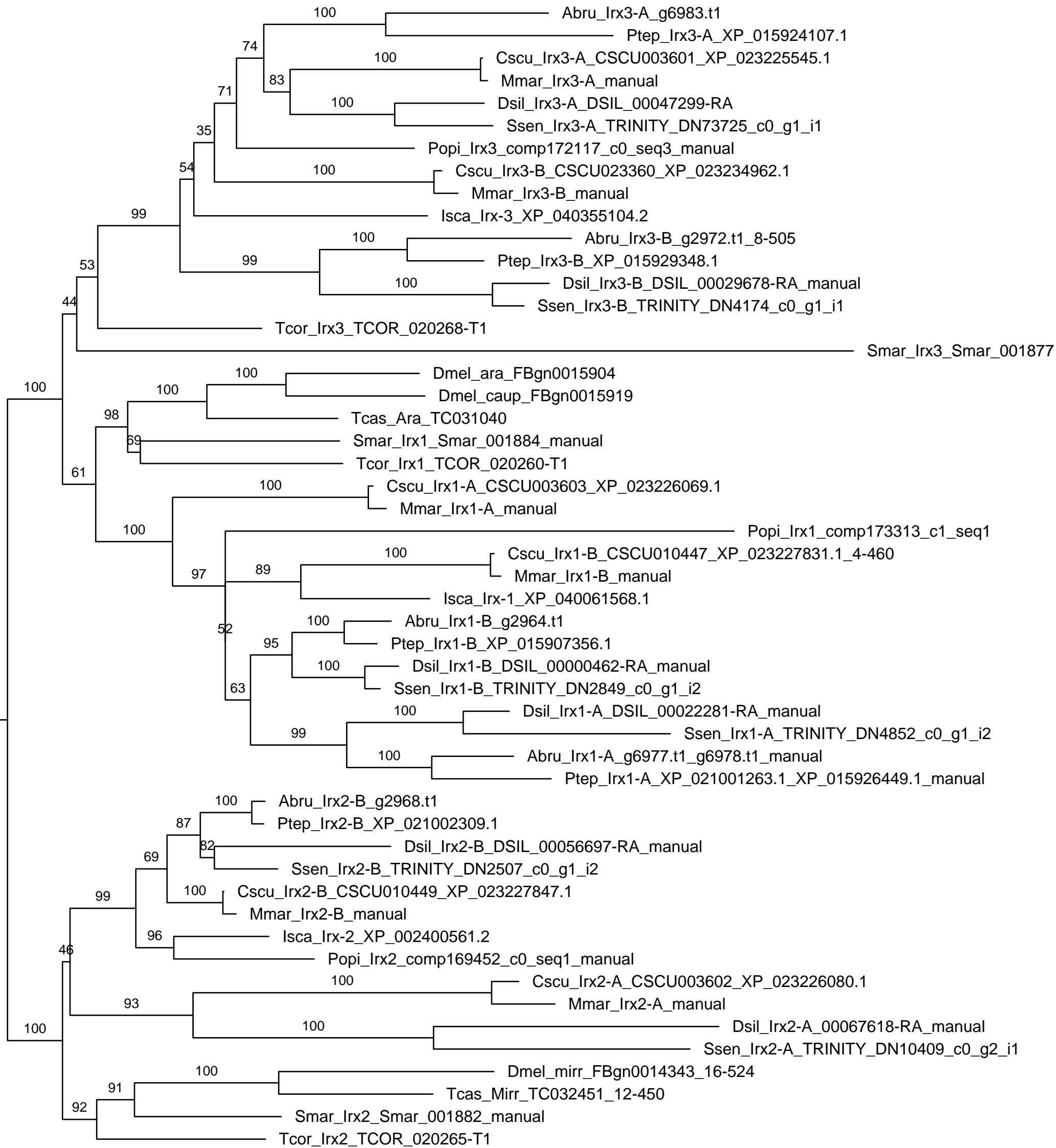

Supplement: msad239_Supplementary_Data [file msad239_supplementary_data.zip › FigureS1.pdf]

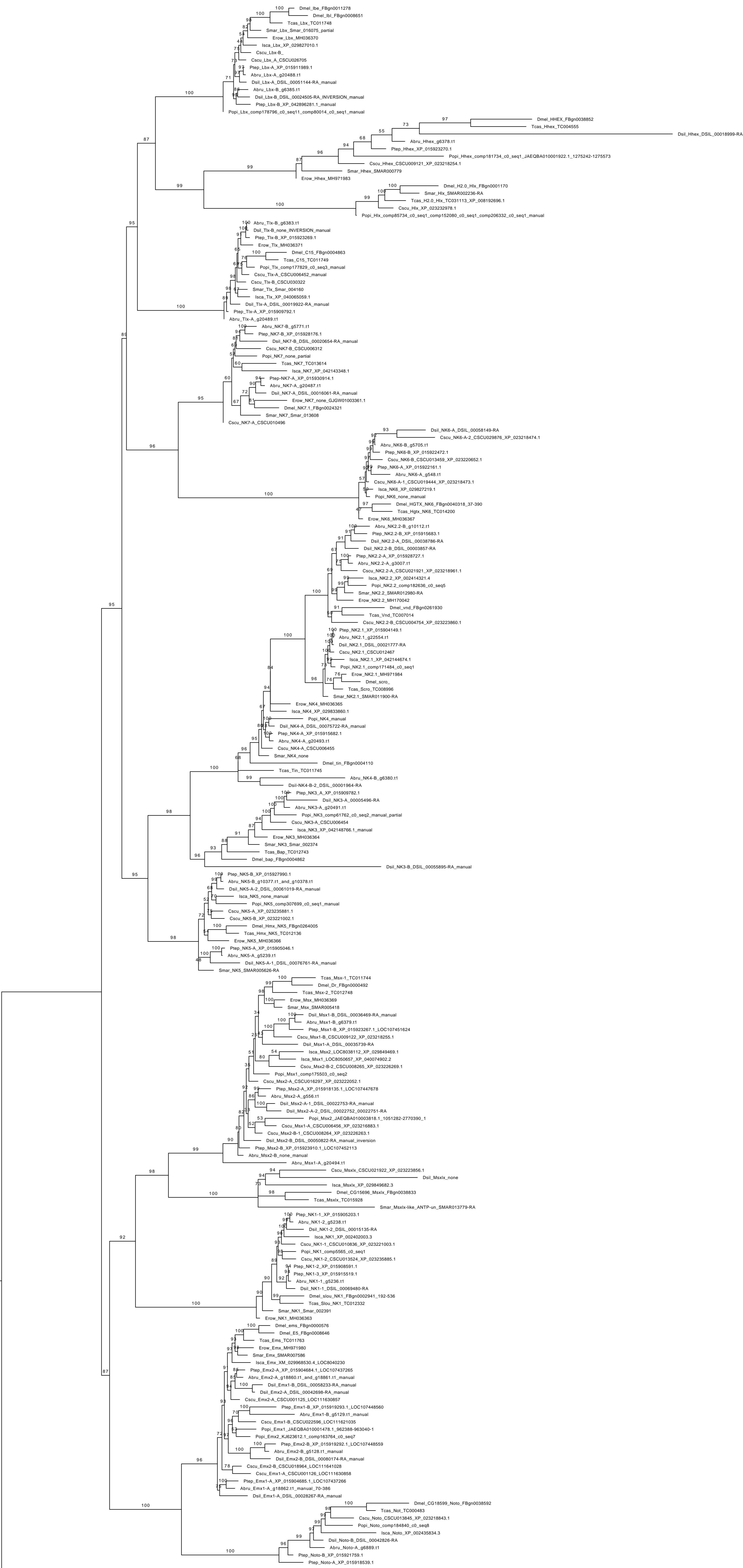

Supplement: msad239_Supplementary_Data [file msad239_supplementary_data.zip › FigureS2.pdf]

**A**

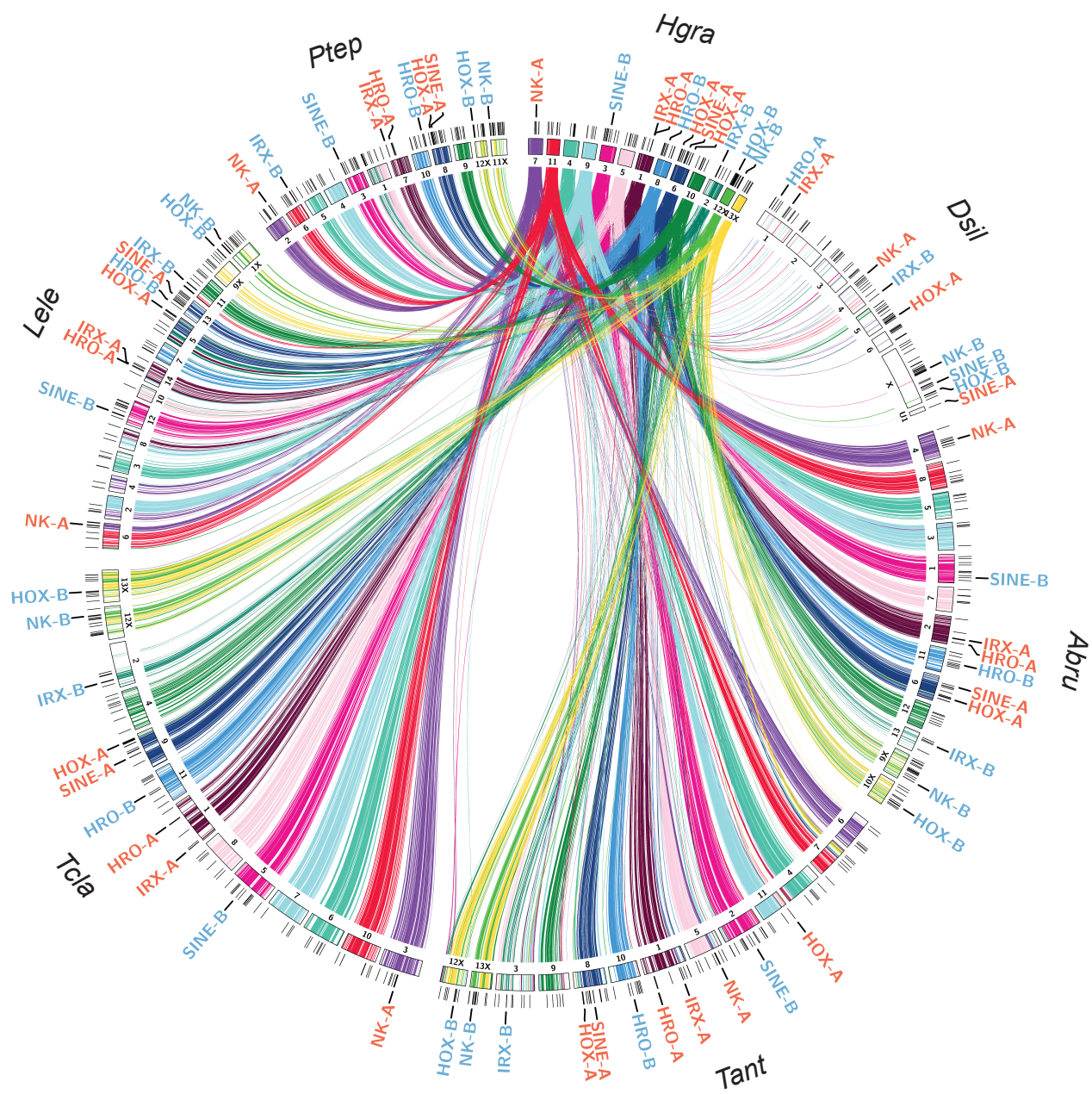

# B

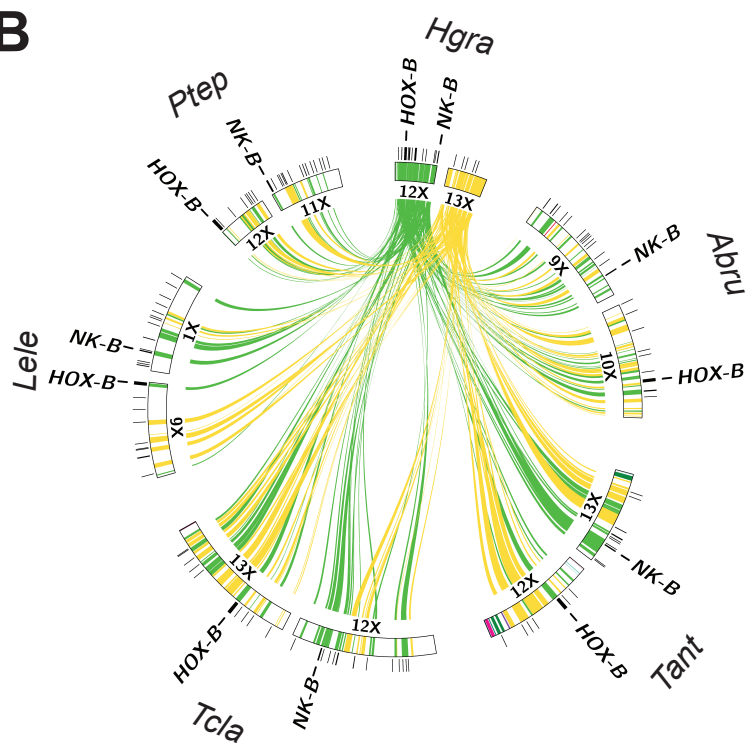

C

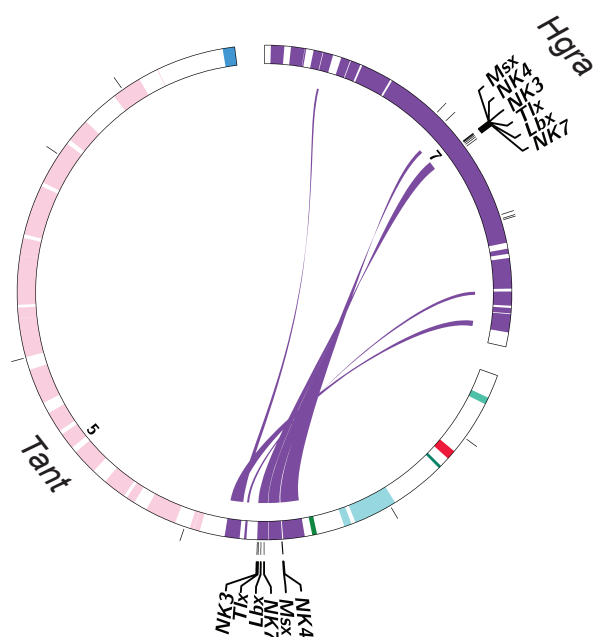

Supplement: msad239_Supplementary_Data [file msad239_supplementary_data.zip › FigureS4.pdf]

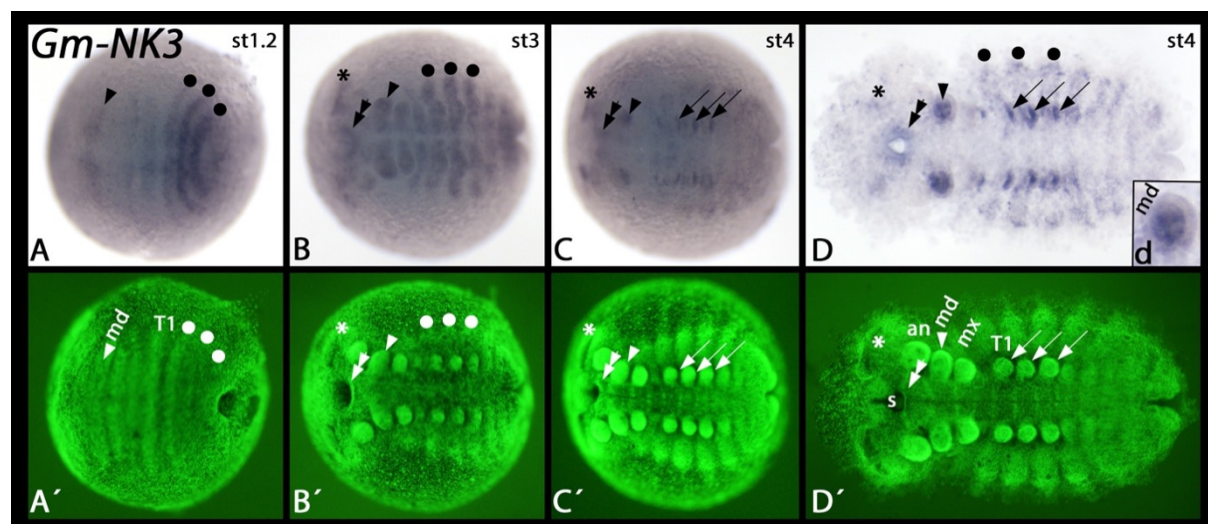

Supplement: msad239_Supplementary_Data [file msad239_supplementary_data.zip › FigureS5.pdf]
